# Formulation Development and Evaluation of Drug Release Kinetics from Colon-Targeted Ibuprofen Tablets Based on Eudragit RL 100-Chitosan Interpolyelectrolyte Complexes

**DOI:** 10.1155/2013/838403

**Published:** 2013-08-06

**Authors:** Kenneth Chibuzor Ofokansi, Franklin Chimaobi Kenechukwu

**Affiliations:** Drug Delivery Research Unit, Department of Pharmaceutics, Faculty of Pharmaceutical Sciences, University of Nigeria, Nsukka 410001, Enugu State, Nigeria

## Abstract

Colon-targeted drug delivery systems (CTDDSs) could be useful for local treatment of inflammatory bowel diseases (IBDs). In this study, various interpolyelectrolyte complexes (IPECs), formed between Eudragit RL100 (EL) and chitosan (CS), by nonstoichiometric method, and tablets based on the IPECs, prepared by wet granulation, were evaluated as potential oral CTDDSs for ibuprofen (IBF). Results obtained showed that the tablets conformed to compendial requirements for acceptance and that CS and EL formed IPECs that showed pH-dependent swelling properties and prolonged the *in vitro* release of IBF from the tablets in the following descending order: 3 : 2 > 2 : 3 > 1 : 1 ratios of CS and EL. An electrostatic interaction between the carbonyl (–CO–) group of EL and amino (–NH_3_^+^) group of CS of the tablets formulated with the IPECs was capable of preventing drug release in the stomach and small intestine and helped in delivering the drug to the colon. Kinetic analysis of drug release profiles showed that the systems predominantly released IBF in a zero-order manner. IPECs based on CS and EL could be exploited successfully for colon-targeted delivery of IBF in the treatment of IBDs.

## 1. Introduction

In recent years, various strategies have been adopted for specific drug delivery to well-defined sites of the gastrointestinal (GI) tract, the colon being the most important one [1–5]. Enteric polymers are used for this purpose, as they are able to release the drug at a particular pH. The pH-sensitive copolymers, such as methacrylic acid/methyl methacrylate copolymers and Eudragit types L and S, dissolve in aqueous media at pH 6 and 7, respectively, which may be equivalent to drug release in the distal ileum [6]. Similarly, chitosan-based polyelectrolyte complexes have been employed as potential carrier materials in drug delivery systems [7]. Furthermore, a growing interest in polyelectrolyte complexes has led to the formulation and characterization of systems involving a variety of anionic and cationic polymers: Eudragit L 30 D-55 and gelatin [8], Eudragit L 100-Eudragit S 100 [9], Eudragit E-Eudragit L [10, 11], Eudragit E-sodium alginate [12], chitosan-alginate/chitosan-carrageenan (mainly kappa-carrageenan with low amounts of lambda-carrageenan) [13], chitosan-polygalacturonic acid [14], chitosan-carboxymethylcellulose [15], and chitosan-alginate [16].

Conventional drug delivery is unfavourable to special cases where drug targeting is applied, that is, when avoidance of gastric dissolution or targeting to the colon is desirable. Colon-targeted drug delivery differs from ordinary enteric coatings (that are designed to merely avoid drug release in the stomach) in that the tablet or capsule is specially formulated to channel greater quantity of drug release to the colonic compartment, thus preventing or highly reducing drug release until the dosage form reaches the colon [17]. Although the large intestine is difficult to access through peroral delivery, it is still favoured as the appropriate site to tackle local colon-related diseases. Colon-targeted delivery could be achieved by the use of pH-dependent systems, time-dependent systems, colonic microflora-activated systems and use of prodrugs [18]. Anti-inflammatory, antibacterial, antiamebic, protein drugs, are a few out of other drugs that can be targeted for site-specific delivery to the colon [19]. 

Ibuprofen is a nonsteroidal anti-inflammatory agent belonging to the group of propionic acid derivatives; it presents a plasmatic half-life of 1.8–2.0 h; as a result, it has to be administered three to six times a day, making this drug a suitable candidate for a controlled release formulation [20]. The swellability properties of IPECs prepared from chitosan (CS) and Eudragit L 100-55 (L 100-55) have been evaluated for their possible pharmaceutical application as new carrier for oral colon-specific drug delivery systems (DDSs) [21]. Similarly, a comparative study of IPECs of chitosan with Eudragit L 100 and Eudragit L 100-55 as potential carriers for controlled oral delivery of diclofenac sodium has been undertaken [22]. However, to the best of our knowledge, there is no scientifically reported study on chitosan-Eudragit RL-100 (CS-EL) polyelectrolyte complexes of ibuprofen. Thus, this study was designed to investigate the formation of IPEC between CS and EL, to characterize the product formed, and to evaluate its performance as a matrix for controlled release of drugs, using ibuprofen (IBF) as a model. 

## 2. Materials and Methods

### 2.1. Materials

Ibuprofen (BASF, Germany), acetic acid, acetone, ammonium acetate, maize starch, magnesium stearate, lactose, concentrated hydrochloric acid (BDH, England), sodium hydroxide (Merck, Germany), and monobasic potassium phosphate (Sigma Chemical Co., USA) were used as purchased from the manufacturers without further purification. All other reagents were of analytical grade and used as such. Distilled water was obtained from an all-glass still. Chitosan of low viscosity nd was fines were retaine (Fluka, Switzerland) and Eudragit RL 100 (MW 135,000) (Rohm Pharma, Germany) were preliminarily dried at 40°C under vacuum for two days.

### 2.2. Preparation of Chitosan: Eudragit RL 100 Interpolyelectrolyte Complexes (IPECs)

 The IPEC of CS and EL was prepared following the standard procedures with slight modifications [12, 23–27]. Chitosan 300 mg was accurately weighed and dissolved in 15 mL of 3% v/v acetic acid followed by the addition of 8 mL volume of 5 M ammonium acetate. Similarly, Eudragit RL 100 (300 mg) was separately dissolved in 7 mL ethanol and was covered to prevent evaporation. This dispersion was slowly added with stirring to the CS solution. The mixture was poured in a Petri plate and was dried at 50°C for 48 h. Films with a total polymer content of 2.5% w/v containing 60 : 40, 50 : 50, and 40 : 60 (i.e., 3 : 2, 1 : 1, and 2 : 3) ratios of chitosan: Eudragit RL 100 were prepared using this method. A control batch (EL) containing only Eudragit RL 100 was also prepared. The dried films were stored in a desiccator until used. 

### 2.3. Preparation of Ibuprofen Granules and Tablets

IBF granules (average weight 297.3 mg) containing 200 mg of IBF were prepared by wet granulation technique [17] using CS : EL interpolymer complexes (50 : 50 w/w) as binder. The damp mass formed was then forced through sieve no. 10 (1.7 mm mesh) and was dried at 50°C for about 1 h until all the moisture was removed. The dry mass was also forced through sieve no. 16 (1.0 mm mesh) and was stored in a desiccator until used. The dried granules were passed through sieve no. 20 and the fines were retained on sieve no. 44. Magnesium stearate (1% w/w) (lubricant) and lactose (bulking agent) were added to the granules. Tablets were compressed using 4 mm biconvex punches in a single station tablet compression machine (Manesty, England) at a pressure of 50 kg/cm^2^. 

### 2.4. Coating of Ibuprofen Tablets

The formulated IBF tablets containing CS : EL (50 : 50 w/w) as binder were coated with aqueous solutions containing (50 : 50, 60 : 40, and 40 : 60 w/w) of CS : EL ratio as IPECs films. The coating solution was sprayed at a rate of 5 mL/min with the help of peristaltic pump using a spray gun of 1 mm nozzle in a coating pan (12′′ diameter) being rotated at 18 rpm. Compressed air was introduced at a pressure of 1.5 kg/cm^2^. The inlet air temperature was maintained at 60°C. The inner surface of coating pan was modified by attaching inert tubes (8 mm diameter) from the centre to the periphery for easy rolling of tablets, thereby ensuring efficient mass transfer of polymer. A control batch coated with Eudragit RL 100 was also prepared.

### 2.5. Swellability of Films Based on Chitosan-Eudragit RL 100 IPECs

The degree of swelling of films of the IPEC was investigated simulating the physiological conditions of the gastrointestinal tract [23–27]. For this purpose, the films were placed in a preweighted basket of the dissolution equipment and immersed for 2 h in 30 mL of 0.1 M hydrochloric acid, then 10 mL of 0.20 M tribasic sodium phosphate was added to pH of 6.8 ± 0.05, and after additional 3 h, another 10 mL of phosphate buffer pH 7.4 was added and the experiment was allowed to continue for another 19 h, giving a total of 24 h. The temperature of the medium was 37 ± 0.5°C. The measurements consisted in removing the basket from the medium, blot-drying by filter paper, and weighing in an analytical balance (Mettler AL 204, Mettler-Toledo Int. Inc., Greifensee, Switzerland). The differences in weight were determined every 30 min.

The degree of swelling was calculated using the formula

(1) $$\begin{array}{c} H\left(\%\right)=\frac{\left(M_{2}-M_{1}\right)}{M_{1}}\times 100, \end{array}$$

where *M*_1_ is the initial weight of the film (g) and *M*_2_ is the final weight of the swollen film (g). The results reported are the mean of three determinations. Degree of swelling at equilibrium and time of swelling were recorded.

### 2.6. Physicochemical Evaluation of the Tablets

#### 2.6.1. Weight Uniformity Test

 Twenty tablets from each batch were weighed together and individually, and the mean weight and percentage deviation were calculated according to British Pharmacopoeia [28].

#### 2.6.2. Friability

 Ten tablets were randomly selected from each batch and weighed. The tablets were set to rotate at 25 rpm for 10 min in an Erweka friabilator. The friability was calculated according to the formula

(2) $$\begin{array}{c} \text{Friability}=\frac{\left(W_{1}-W_{2}\right)}{W_{1}}, \end{array}$$

where *W*_1_ is the initial weight and *W*_2_ is the final weight. 

#### 2.6.3. Crushing Strength/Hardness

 Ten tablets from each batch were randomly selected. The force required to break each tablet was determined using a Monsanto-Stokes tablet tester. The average force of the ten tablets was taken as the crushing strength (kgf).

#### 2.6.4. Disintegration Time

 This test was carried out using a method already described [29]. Three tablets were randomly selected from each batch and were placed in the inner compartment of a disintegration apparatus (which was tied with a thermoresistant thread to the clamp of a retort stand) of the disintegrating apparatus containing 500 mL of distilled water maintained at 37 ± 1°C. The medium was stirred at 150 rpm and the time taken for the tablets to disintegrate was recorded. The test was performed in triplicate for each batch, and the average time for each batch was calculated.

#### 2.6.5. *In Vitro* Drug Release Studies

*In vitro* release of ibuprofen from the tablets was performed using USP (Dissolution Apparatus 1-basket method) at 37 ± 0.5°C and 100 rpm in three release media (pH 1.2, 6.8, and 7.4). Each tablet was placed in the cylindrical basket of a dissolution apparatus (Veego, India) attached to the rotating spindle suspended in the dissolution medium of volume of 900 mL (pH 1.2). The rectangular glass container into which the one-litre cylindrical plastic container was immersed was filled with sufficient water to get more than half of the cylindrical container immersed in the water. The heating element in it was switched on and allowed to equilibrate at a temperature of 37 ± 0.5°C. The equipment was switched on to rotate at a speed of 100 rpm. At predetermined time intervals, 5 mL samples of the dissolution medium were withdrawn and were assayed spectrophotometrically (UV/VIS, Unico, USA) after appropriate dilution and filtration. Meanwhile, 5 mL of a fresh medium was used to refresh the dissolution medium. The dissolution was first run for 2 h in medium of pH 1.2. Two hours were chosen to mimic the average gastric emptying time [17]. At the end of the 2 h, the equipment was switched off the rotating spindle attached to the basket-bearing tablet was unscrewed out and properly rinsed of the previous medium after carefully removing the tablet. The cylindrical plastic material containing the dissolution medium was also disposed of the pH 1.2 medium and adequately rinsed with purified water. Then, 900 mL of a second dissolution medium, pH 6.8, was emptied into the 900 mL plastic container and the temperature allowed to attain 37 ± 0.5°C. Then, the tablet was reinstated in the basket attached to the spindle. The spindle was screwed back in place and dissolution run as before but for 3 h. The average time for change of dissolution medium was about 20 min. Three hours was chosen because the reported average intestinal transit time is 3-4 h [17]. At the end of 3 h, the medium was again removed and replaced with a third medium of pH 7.4 to mimic the ileocecal pH [17] and the same process was repeated but this time until the tablet released all or nearly all the drug. Three replicate tests were carried out. Previous studies indicate that polymers did not interfere with the determination of the model drug, IBF [23–27].

The withdrawn samples were immediately analyzed using a spectrophotometer at 221 nm, 272 nm, and 281 nm for the release study in the pH 1.2, 6.8, and 7.4 medium, respectively.

## 3. Results and Discussion

Administration of NSAIDs such as ibuprofen is usually associated with gastrointestinal disturbances [12, 23–27]. Thus, research efforts have been directed to solve, or at least improve, this inconveniences, through various techniques of protection of the gastric mucosa or alternatively of preventing the release of NSAIDs in the gastric region. The site-specific delivery of drugs to the colon can be highly advantageous for various applications including the local treatment of inflammatory bowel diseases (IBDs). In this study, various IPECs, formed between EL and CS, were obtained and evaluated as potential colon-targeted oral controlled release matrices for IBF, a model NSAID. The IPECs films were formulated by nonstoichiometric method, and tablets containing IBF and IPECs were prepared by wet granulation technique. The formulations were evaluated in terms of friability, hardness, disintegration, swellability, and drug dissolution. Here, the liquid ethanol was employed for dissolving the EL so as to enable its proper incorporation into the CS to form the IPECs. Lactose was selected as the bulking agent, maize starch as the disintegrant, and magnesium stearate as the lubricant.

### 3.1. Characterization of Chitosan-Eudragit RL 100 IPEC-Based Ibuprofen Tablets

 The tablets were smooth in appearance, circular in shape and whitish in colour. The mean weight of the various batches of the tablets (Table 1) ranged from 296.32 ± 0.30 mg to 301.57 ± 0.93 mg. This shows that all the batches met compendial requirement for weight variation [28, 29], implying that these tablets were uniform in weight. Table 1 equally indicates that average times of 55.97 ± 2.84, 70.25 ± 1.63 and 60.81 ± 3.87 min each was required for tablets containing respectively 1 : 1, 2 : 3 and 3 : 2 ratios of CS and EL, and 35.79 ± 2.45 min for EL only-based tablets to disintegrate at the experimental conditions. This (disintegration time) test was performed to determine the ease with which IBF is released from the tablets at a controlled temperature of 37 ± 1°C. The results indicate that the adhesive force existing between the components of the tablets of batch 2 : 3 is more than that in the tablets of batch 3 : 2 and lowest in the tablets of batch 1 : 1. The reason for this is uncertain, but may be attributed to greater concentration of the EL on the tablets of batch 2 : 3 than tablets of batch 3 : 2. This implies that EL exerted significant effect on the force of adhesion of the tablet ingredients, thereby increasing the disintegration time. The data equally revealed that the tablets of batches 2 : 3 and 3 : 2 demonstrated greater sustained release effect than tablets of batch 1 : 1. It is also likely that high concentration of EL and CS in the tablets of batch 2 : 3 and 3 : 2, respectively, was responsible for this. More so, the low disintegration time of the tablets of batch 3 : 2 suggests that these tablets have prospects of dose dumping. Furthermore, the friability test was carried out to determine the ability of the tablets to withstand mechanical shock or abrasion. Low values of friability indicate high resistance to abrasion and good binding/adhesion properties [28, 29]. The friability test result revealed that all the batches met compendial requirement for resistance to abrasion, with tablets of batch CS : EL (2 : 3) and batch EL having the greatest (0.95 ± 0.01%) and least (0.72 ± 0.03%) resistance to abrasion, respectively. In addition, the crushing strength test was undertaken to determine the level of resilience of the tablets to crushing when a force is applied. The crushing strength results show that tablets of batch CS : EL (2 : 3) possessed the highest mean crushing strength of 4.71 ± 0.32 kgf followed by tablets of batch CS : EL (1 : 1), which is 4.62 ± 0.09 kgf. The lowest crushing strength of 4.15 ± 0.27 kgf was observed in tablets of batch EL. The implication is that tablets of batch CS : EL (2 : 3) have higher adhesive force than tablets of batch CS : EL (1 : 1), and this force holds the components of these tablets together such that they are not easily broken. Tablets of batch EL have the least force of adhesion, and thus these tablets are easily broken. For compressed tablets, a crushing strength ≥5 kgf is considered the upper limit of acceptance and since none of the batches of the tablets exceeded this value; then they are acceptable.

### 3.2. Swellability of the IPECs Film

 It is well known that the potential of polymeric carriers to be used as controlled release materials can be predicted by determination of their swelling characteristics [22]. In a previous study, a group of researchers evaluated the swelling behavior of polycomplex matrices made from CS and EL 100 in simulated gastro-intestinal tract (GIT) and all systems used were stable in pH 1.2 (1 h) and pH 6.8 (2 h) [26]. According to the specifications of Degussa, the dissolution of EL depends on the copolymer structure and is well regulated by the ratio between methyl methacrylate or ethyl acrylate and methacrylic acid. 

**Figure 1:**
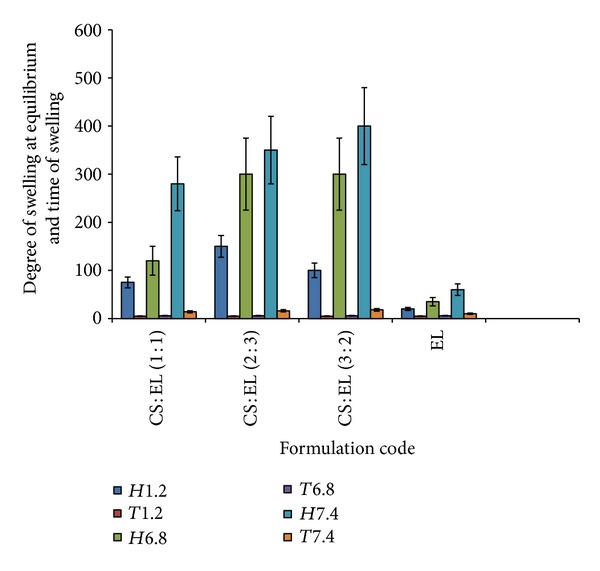
The various degrees of swelling at equilibrium (*H*%) and the time of swelling (*T*) at media pH 1.2, 6.8, and 7.4, respectively. CS : EL (1 : 1) = tablets coated with IPEC containing 50% chitosan and 50% Eudragit RL 100. CS : EL (2 : 3) = tablets coated with IPEC containing 40% chitosan and 60% Eudragit RL 100. CS : EL (3 : 2) = tablets coated with IPEC containing 60% chitosan and 40% Eudragit RL 100. EL = control tablets coated with IPEC containing only Eudragit RL 100.

Figure 1 shows degree of swelling at equilibrium and time of swelling for the different IPEC films. In Figure 1, *H*1.2 and *T*1.2 represent the degree of swelling at equilibrium at pH 1.2 and the time of swelling, respectively. Similarly *H*6.8 and *T*6.8 also represent the degree of swelling at equilibrium at pH 6.8 and the time of swelling, respectively. The EL only-based films showed a marginal swelling in the media used. The swelling profiles are similar: increasing degree of swelling in acidic medium due to a progressively increasing number of ionized –NH_3_^+^ groups of CS and decreased swellability for systems containing EL, probably due to leaching of undissolved particles of EL. The swelling behavior of IPECs films is completely different from that of the EL only-based films (Figure 1). In these systems, the electrostatic repulsion of free ionized amino groups is responsible for swelling. In case of IPEC made up of CS : EL 2 : 3, the degree of swelling was 150% at pH 1.2, but afterwards a two-fold increase in swelling at pH 6.8 could be observed.

 On immersing the polycomplex matrix into the acidic medium (pH 6.8), free amino groups got protonated and their hydration increased the degree of swelling within the first part of the experiment. Later, full ionization of all amino groups turned it into a polyelectrolyte with a relatively high charge density. As a result, the structure of the IPEC is changed because the ionic bonds are not fixed and they could move from one electrostatic site to another [30, 31]. The protonated carboxylic acid groups of EL (weak polyacid) became charged by ionized amino groups of CS to form new interpolymer contacts. Comparable observations were made with IPEC prepared from two types of Eudragit [9–11] and Eudragit E 100 and alginate sodium [12]. However, the degree of swelling was much higher for the current IPECs.

 After transferring the matrix to the second medium of pH 6.8, carboxylic groups of EL became more ionized giving rise to an increase in the degree of swelling. However, previously protonated amino groups began to lose their charge and may be responsible for the increase in the hydrophobic units in the IPEC structure. As a result, the swelling slightly decreased at the end of the second medium (pH 6.8) but began to increase in the third buffer (pH 7.4) due to a progressive increase in the number of carboxylate group, in spite of the solubility of CS which decreased at higher pH values.

The formulated IPECs, as many of the investigated stoichiometric polycomplexes, would have a more or less homogenous network structure in the swollen state, which could be changed during swelling. This structure is clearly sensitive to pH changes. Completely different changes were observed in the CS : EL (3 : 2) system. The polycomplexes showed the highest degree of swelling; increasing the CS content led to an increase in the swellability of the IPECs. This system is stable in the first acidic medium, but with a relatively low degree of complexation, and completely destructive to individual polymers afterwards. This system is very sensitive to pH and is not stable in simulated intestinal tract (SIT) conditions. The reason is that polycomplexes with participation of EL (consisting of more hydrophobic methacrylate chains) are simply destroyed in neutral media. Similar results of high pH sensitivity were observed in polycomplex systems made up of CS-pectin [32] and CS-dextran sulfate [33].

### 3.3. Drug Release Studies

**Figure 2:**
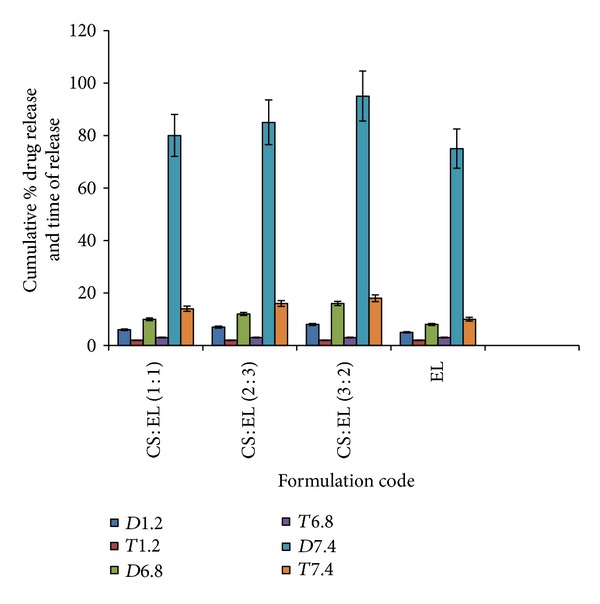
The various cumulative % drug release (*D*) and the time of release (*T*) at media pH of 1.2, 6.8, and 7.4, respectively. CS : EL (1 : 1) = tablets coated with IPEC containing 50% chitosan and 50% Eudragit RL 100. CS : EL (2 : 3) = tablets coated with IPEC containing 40% chitosan and 60% Eudragit RL 100. CS : EL (3 : 2) = tablets coated with IPEC containing 60% chitosan and 40% Eudragit RL 100. EL = control tablets coated with IPEC containing only Eudragit RL 100.

In order to assess the potential of the IPECs to be used in matrix controlled drug delivery systems, we evaluated the release of the model drug (IBF) from all investigated matrix systems. Based on the results of the previous studies from dissolution behavior of IBF, as a model drug, from the polycomplex matrix systems based on CS and EL in gastrointestinal simulated conditions [26], we decided to use three release media (pH 1.2, 6.8, and 7.4) in the present study. The polycomplexed matrices are stable in gastric simulated environment. IBF is insoluble at pH 1. In Figure 2, *D*1.2 and *T*1.2 represent the cumulative amount of drug released at pH 1.2 and the time of release respectively. Similarly, *D*6.8 and *T*6.8 also represent the cumulative amount of drug released at pH 6.8 and the time of release, respectively. The same thing is applicable for pH 7.4. 

 As expected, very low IBF release occurred in a pH-gradient (from 6.8 to 7.4) medium showing that, below solubility of the enteric copolymers, no drug release occurred (EL). After this lag time, the drug release occurred continuously. As shown in Figure 2, polycomplex matrices made up of CS and EL showed a release behavior that is somehow slower than that of the tablets coated with only EL. The reason is that due to high swelling properties at all pH values, these polycomplexes form gel-like matrices, which can sustain IBF release. In case of CS : EL (3 : 2) polycomplex, the release of IBF was slowest, with the most constant drug release rate as well as swelling properties when compared to all the other systems. This means that an excess of CS in the IPEC structure led to formation of a well-equilibrated polycomplex (with a high degree of complexation) which is not so pH sensitive and stable in SIT conditions. 

 It is evident that general retardation and low amount of drug release took place at pH 1.2 and 6.8, respectively; that is, all the IPECs batches released negligible quantity of drug in the first two dissolution media when compared to pH 7.4. It has been reported that a successful colon-targeted delivery system should be able to retard or withhold drug release in the upper part of the gastrointestinal region but release the drug promptly on entry into the colon [34], since the pH gradient ranges from 1.2 in the stomach through 6.6 in the proximal small intestine to a peak of up to 7.5 in the distal small intestine [35]. The control batch (EL) coated with only EL recorded the lowest cumulative drug release at both pH 1.2 and pH 6.8. However, a closer look showed that the cumulative percent drug release (*D*7.4) at pH 7.4 for the three (IPEC) batches were between 80 and 95%. This means that it has the potential of making sufficient quantity of drug available in the colon, but then how long (*T*7.4) it would take the drug to be released in the colon is more important.

Although it is good for a colon-specific delivery system to withhold drug release at both pH 1.2 and 6.8 for some reasonable hours, but being able to promptly release drug at the ascending colon, it is much better if release spreads throughout the colon. The longer the time (*T*7.4) the higher the probability that release would continue all through the colonic transit period. The IPECs presented the possibility of having a greater contact time in the colon, greater duration of action, and larger area of action. It has been reported that gastrointestinal (GI) absorption of orally administered drugs is determined by not only the permeability of GI mucosa but also the transit rate in the GI tract [36]. This envisaged that improved drug release by the IPECs may likely cause the IBF to impinge on infected cells as in colitis and colorectal cancer, consistent with an earlier report [37]. Overall, the release profiles of the tablets based on the IPECs are characterized by a constant and slow release behavior (sustained-release systems). More so, the release profiles are in agreement with the results obtained in the swelling studies.

It is pertinent to draw attention to some advantages of our coated tablets, which have sustained release property may have in common with multiparticulate dosage forms. Actually some reporters have favoured multiparticulate dosage forms as presenting better advantages over single dosage forms. This is because the use of single unit dosage forms for colon-targeted delivery has been found to be fraught with some shortcomings such as premature disintegration due to production flaws or sudden change in GIT physiology, which could lead to reduced bioavailability or therapeutic efficacy. On the other hand, some advantages of multiparticulate dosage forms for colon targeting include reduced risk of systemic toxicity, increased bioavailability, low propensity to cause local irritation, and predictable gastric emptying [38]. Our dosage form design is composed of multiparticulates within a unit dosage form from where gradual release took place. This may likely enable the coated tablets to enjoy many if not all the advantages of multiparticulates enumerated previously. 

### 3.4. Kinetics of Drug Release

In order to understand the mechanism and kinetics of drug release, the results of the *in vitro *drug release study were fitted into various kinetic equations like zero order (cumulative percent drug released versus time), first order (log cumulative percent drug retained versus time), Higuchi (cumulative percent released versus $\sqrt{T}$), and Peppas (log of cumulative percent drug released versus log time) as depicted in Table 2. The kinetic model that best fits the dissolution data was evaluated by comparing the coefficient of determination (*r* ^2^) values obtained in various models. In the Peppas (Fickian diffusion) model, mechanisms of drug release are characterized using the release exponent (“*n*” value). An “*n*” value of 1 corresponds to zero-order release kinetics (case II transport); 0.5 < *n* < 1 means an anomalous (non-Fickian) diffusion release model; *n* = 0.5 indicates Fickian diffusion, and *n* > 1 indicates a super case II transport relaxational release [39].

 Results of the kinetic analysis of drug release (Table 2) indicates that the most predominant release mechanism was zero order. This was corroborated by its Ritger-Peppas “*n*” values of between 1.00 and 1.16 which implies super case II release kinetics [40] (a strong indication of zero order). Zero-order release is the ideal in controlled drug release and has been reported not to be common with matrix systems, this being attributed to time-dependant changes in drug depleted matrix surface area and diffusional path length [17]. Therefore, to achieve linear or zero-order release with matrix systems, several manipulative strategies would be inevitably required to impart geometric and structural adjustments on the tablets [41–43]. Zero-order release has a lot of advantages including ability to deliver drug at a constant rate, thus providing a predictable bioavailability status.

## 4. Conclusions

 In this study, IPECs were formed between CS and EL. This changed the structure of the polyelectrolytes and could regulate their properties. The differences between the different IPECs that were observed during the swelling experiments as well as during the drug release studies show that drug release could be tuned based on the composition of the IPEC, with CS : EL (3 : 2) IPECs as the best formulation. This study has shown that IPECs based on CS and EL could be exploited successfully for colon-targeted delivery of IBF in the treatment of IBDs.

## Figures and Tables

**Table 1:** Physicochemical properties of the tablets.

Formulation code	Average friability (%)^¶,||^	Mean weight (mg)^¶,§^	Mean crushing strength (kgf)^¶,#^	Average disintegration time (min)^¶,§^
CS : EL (1 : 1)	0.91 ± 0.02	297.03 ± 1.07	4.62 ± 0.09	55.97 ± 2.84
CS : EL (2 : 3)	0.95 ± 0.01	301.57 ± 0.93	4.71 ± 0.32	70.25 ± 1.63
CS : EL (3 : 2)	0.86 ± 0.01	299.62 ± 0.84	4.34 ± 0.18	60.81 ± 3.87
EL	0.72 ± 0.03	296.32 ± 0.30	4.15 ± 0.27	35.79 ± 2.45

^¶^Mean ± SD, ^§^ *n* = 20, ^#^ *n* = 3, and ^*||*^ *n* = 10.

CS : EL (1 : 1): tablets coated with IPEC containing 50% chitosan and 50% Eudragit RL 100.

CS : EL (2 : 3): tablets coated with IPEC containing 40% chitosan and 60% Eudragit RL 100.

CS : EL (3 : 2): tablets coated with IPEC containing 60% chitosan and 40% Eudragit RL 100.

EL: control tablets coated with IPEC containing only Eudragit RL 100.

**Table 2:** The various release models and their release parameters.

Batch code	Higuchi		Zero-order		First-order		Ritger-Peppas	
	*K*_*H*_	*R* ^2^	*K*_0_	*R* ^2^	*K*_*F*_	*R* ^2^	*N*	*R* ^2^
CS : EL (1 : 1)	14.72	0.888	9.68	0.996	−0.13	0.918	1.12	0.763
CS : EL (2 : 3)	26.90	0.917	13.59	0.997	−0.20	0.731	1.16	0.825
CS : EL (3 : 2)	34.18	0.837	10.46	0.999	−0.09	0.816	1.05	0.855
EL	19.57	0.831	6.82	0.995	−0.05	0.777	1.10	0.773

*K*_0_: zero-order release rate constant; *K*_1_: first-order release rate constant; *K*_*H*_: higuchi release rate constant; *R* ^2^: regression line value; *n*: Ritger-Peppas value.

CS : EL (1 : 1): tablets coated with IPEC containing 50% chitosan and 50% Eudragit RL 100.

CS : EL (2 : 3): tablets coated with IPEC containing 40% chitosan and 60% Eudragit RL 100.

CS : EL (3 : 2): tablets coated with IPEC containing 60% chitosan and 40% Eudragit RL 100.

EL: control tablets coated with IPEC containing only Eudragit RL 100.

## Abbreviations

IBF: Ibuprofen
IBDs: Inflammatory bowel diseases
CS: Chitosan
EL: Eudragit RL 100
IPECs: Interpolyelectrolyte complexes
NSAIDs: Nonsteroidal anti-inflammatory agents
DDS: Drug delivery systems
GIT: Gastrointestinal tract.
